# Prognostic implications of right ventricular to pulmonary artery uncoupling in cardiac amyloidosis

**DOI:** 10.3389/fcvm.2025.1653950

**Published:** 2025-09-29

**Authors:** Aiste Monika Jakstaite, Anja Hänselmann, Samira Soltani, Eleonora Angelini, Michael Heuser, Vega Gödecke, Stefan Gingele, Thomas Skripuletz, Johann Bauersachs, Udo Bavendiek, Dominik Berliner

**Affiliations:** ^1^ Department of Cardiology and Angiology, Hannover Medical School, Hannover, Germany; ^2^ Amyloidosis Centre Lower Saxony, Hannover Medical School, Hannover, Germany; ^3^ Department of Internal Medicine IV, University Hospital Halle, Martin Luther University Halle-Wittenberg, Halle, Germany; ^4^ Department of Hematology, Hemostasis, Oncology and Stem Cell Transplantation, Hannover Medical School, Hannover, Germany; ^5^ Department of Nephrology and Hypertension, Hannover Medical School, Hannover, Germany; ^6^ Department of Neurology, Hannover Medical School, Hannover, Germany

**Keywords:** right ventricular–pulmonary arterial coupling, amyloidosis, transthyretin amyloidosis, heart failure, speckle-tracking echocardiography

## Abstract

**Background:**

Right ventricular–pulmonary arterial (RV–PA) uncoupling in cardiac amyloidosis (CA) has been underexplored, with focus mainly on tricuspid annular plane systolic excursion (TAPSE)/pulmonary artery systolic pressure (PASP). This study aims to evaluate the association of various echocardiographic surrogates of RV–PA coupling with outcomes in cardiac transthyretin (ATTR-CA) and light-chain (AL-CA) amyloidosis.

**Methods:**

We analyzed RV–PA coupling in patients diagnosed with ATTR-CA and AL-CA at our center between 2014 and 2023. RV–PA coupling was assessed using TAPSE/PASP, fractional area change (FAC)/PASP, and RV free wall strain (RVFWS)/PASP. The primary endpoint was all-cause mortality.

**Results:**

A total of 120 patients (86% ATTR-CA, 14% AL-CA) were included in the study (median age 77 years, 88% male). During a median follow-up period of 23 (IQR: 15–34) months, the primary endpoint occurred in 25 patients (21%). The study population was stratified based on the ROC-derived TAPSE/PASP cutoff of <0.30 mm/mmHg, demonstrating RV–PA uncoupling. Lower RV–PA coupling surrogates were independently associated with higher mortality (HR per +0.1 unit: TAPSE/PASP, 0.74, 95% CI: 0.59–0.93, *p* = 0.011; FAC/PASP, 0.87, 0.77–0.98, *p* = 0.018; RVFWS/PASP, 0.78, 0.63–0.97, *p* = 0.024). TAPSE/PASP demonstrated the strongest prognostic discrimination (AUC: 0.79, bootstrapped 95% CI: 0.66–0.91), compared with FAC/PASP (AUC: 0.75, 0.58–0.91) and RVFWS/PASP (AUC: 0.72, 0.52–0.87).

**Conclusions:**

RV–PA uncoupling may be linked to a higher risk of all-cause mortality in CA. TAPSE/PASP outperformed numerically FAC/PASP and RVFWS/PASP in predicting long-term survival, although it did not clearly outperform established RV function parameters.

## 1 Introduction

Cardiac amyloidosis (CA) is an increasingly recognized cardiomyopathy caused by the extracellular accumulation of misfolded proteins, in most cases involving immunoglobulin light chains (AL) or transthyretin (ATTR) (1–4). AL amyloidosis, particularly when diagnosed at an advanced stage, is associated with poor outcomes and can progress rapidly. In contrast, ATTR-CA typically follows a more gradual disease course. Nevertheless, novel prognostic markers are needed given the latest treatment developments for both AL and ATTR-CA. CA, especially in the latter stages, encompasses a restrictive filling pattern, leading to elevated filling pressures (5). Right ventricular (RV) amyloid infiltration is an important pathophysiological contributor to RV dysfunction. In addition, the transmission of increased pressures to the pulmonary vasculature results in secondary pulmonary hypertension (PH) and contributes to RV dysfunction, which independently predicts outcomes in patients with CA (6–8). RV longitudinal strain is a more sensitive indicator of RV dysfunction than traditional echocardiographic parameters and can aid in revealing occult RV dysfunction (6, 9, 10). More recently, attention has been drawn to the RV to pulmonary artery (PA) coupling, which refers to the relationship between RV contractility and its afterload, offering a more physiology-based insight into RV function. While the measurement of the end-systolic/arterial elastance (Ees/Ea) ratio from invasive pressure–volume loops provides the most accurate assessment of RV–PA coupling, focus has recently shifted toward the non-invasive surrogate parameters (11, 12). The ratio between tricuspid annular systolic excursion (TAPSE) and pulmonary systolic pressure (PASP) correlates well with the invasively measured Ees/Ea ratio (13–15). The uncoupling between RV and PA, as assessed by the tricuspid annular plane systolic excursion (TAPSE)/pulmonary artery systolic pressure (PASP) ratio, was associated with poor outcomes in diverse patient cohorts with heart failure (HF) and valvular heart disease (16–19). In addition to the widely used TAPSE/PASP ratio, the RV free wall strain (RVFWS)/PASP and fractional area change (FAC)/PASP ratios have also been evaluated as non-invasive surrogates of RV–PA coupling in recent studies (1). The TAPSE/PASP ratio has been shown to outperform TAPSE or PASP alone in predicting outcomes in both HFrEF and HFpEF populations (2). Similarly, in a cohort of patients with pulmonary arterial hypertension, the RVFWS/PASP ratio demonstrated stronger prognostic value than RVFWS alone (3). The RV–PA uncoupling in CA has been underexplored, with analyses primarily focusing only on TAPSE/PASP (20–22). It is unclear whether alternative parameters of RV function, such as afterload-corrected RVFWS and FAC, would outperform the prognostic value of TAPSE/PASP in patients with CA. The present study aimed to investigate echocardiographic RV–PA coupling surrogates, including the TAPSE/PASP, RVFWS/PASP, and FAC/PASP ratios, and to compare their association with outcomes in patients with AL-CA and ATTR-CA.

## 2 Materials and methods

### 2.1 Study population

This study retrospectively included consecutive patients from Hannover Medical School diagnosed with AL-CA or ATTR-CA from 2014 to 2023. The diagnosis of CA was established when AL or ATTR amyloid was detected in an endomyocardial biopsy or in an extracardiac biopsy, supported by characteristic imaging findings indicative of CA. More recently, a non-invasive approach has been employed, and the diagnosis of ATTR-CA was made in cases exhibiting Perugini grade 2 or 3 uptake in bone scans, along with normal serum free light chains (FLC), FLC ratio, and the absence of monoclonal protein (4). Clinical data were collected from the individual electronic health records obtained within the clinical routine. Follow-up was conducted at least annually as part of the clinical routine. Patients with poor echocardiographic image quality, including inadequate visualization of the RV and poor continuous wave Doppler signal for PASP estimation, as well as those lacking follow-up data, were excluded from the analysis. The study conforms with the principles outlined in the Declaration of Helsinki and received approval from the local ethics committee (11770-B0-K-2025).

### 2.2 Echocardiography and speckle-tracking strain analysis

Transthoracic echocardiography was performed using Philips iE33, EPIQ 7, or EPIQ CVx (Philips Electronics, Eindhoven, The Netherlands). All echocardiographic measurements were performed in accordance with the guidelines (5, 6). In patients with atrial fibrillation, echocardiographic parameters were obtained by averaging three consecutive cardiac cycles. Left ventricular ejection fraction (LVEF) was calculated using Simpson's biplane method from apical two- and four-chamber views. The following RV parameters were analyzed: RVFWS (performed offline), TAPSE, FAC (performed offline), maximal tricuspid regurgitation velocity (TRV_max_), and estimated PASP. TAPSE was measured within M-mode intersecting the lateral tricuspid annulus. FAC was measured offline by tracing the RV endocardial borders at end-diastole and end-systole in the apical four-chamber view with focus on the RV. PASP was estimated by measurement of TRV_max_ and right atrial pressure (RAP): PASP = 4(TRV_max_)^2^ + RAP. RAP was estimated from the inferior vena cava (IVC) diameter: IVC diameter ≤21 mm with >50% collapse during sniffing suggesting normal RA pressure (0–5 mmHg), IVC diameter ≤21 mm with <50% collapse during sniffing or >21 mm with >50% collapse suggesting RA pressure of 5–10 mmHg, and IVC diameter >21 mm with <50% collapse during sniffing suggesting RA pressure of ≥15 mmHg (7). The TOMTEC Image Arena TTA 2.21.03/ISCV TOMTEC Integration TTA2.41.00 software (TOMTEC Imaging Systems GMBH, Unterschleissheim, Germany) was used for the offline speckle-tracking strain and FAC analysis. The peak longitudinal systolic strain of the RV free wall and interventricular septum was measured from an apical four-chamber view with focus on the RV. Strain analysis was performed in patients with adequate RV endomyocardial border definition that was traced automatically by the software after setting the reference points and adapted manually for optimal tracking throughout the cardiac cycle. The RVFWS is automatically divided by the software into basal, middle, and apical segments. The averaged, software-generated mean value of RVFWS was used for the analysis.

RV–PA coupling was assessed using three different echocardiographic surrogates: TAPSE/PASP, FAC/PASP, and RVFWS/PASP ratios. A receiver operating characteristic (ROC)-derived TAPSE/PASP cutoff was used to identify RV–PA uncoupling and stratify the population.

### 2.3 Outcomes

The primary outcome of the study was all-cause death. The follow-up period was calculated as the interval between the first presentation at Hannover Medical School and the last available follow-up date or the date of death. Outcome data were collected during follow-up visits within clinical routine and from clinical records. In a few cases where this information was missing, patients were contacted directly, ensuring that mortality status was known for all patients included in the study.

### 2.4 Statistical analysis

Continuous variables are demonstrated as mean ± standard deviation or as median [interquartile range (IQR)], depending on the presence of a normal distribution, and categorical variables as counts and percentages. Continuous data were evaluated for normality of distribution with the Shapiro–Wilk test. A two-sided *t*-test was used for comparison of continuous, normally distributed data, and the non-parametric Mann–Whitney *U* test for non-normally distributed data. Associations between categorical variables were tested using the chi-square (*χ*^2^) test. Survival analysis was performed by the Kaplan–Meier method, with between-group differences assessed by the log-rank test. The prognostic impact of RV–PA coupling parameters (TAPSE/PASP, FAC/PASP, RVFWS/PASP) was assessed with univariable Cox proportional hazards regression models. Due to the low number of events, multivariable analysis was not feasible. To derive clinically relevant thresholds, we performed a fixed-time ROC analysis at 24 months, classifying patients who died within 24 months as cases and those alive with ≥24 months of follow-up as controls. The Youden index was applied to determine optimal cutoffs for each parameter, and diagnostic performance was summarized by AUC, sensitivity, and specificity with bootstrap 95% confidence intervals. *p* < 0.05 was considered statistically significant. Analyses were performed using SPSS version 29 (IBM Corp., Armonk, NY, USA) and GraphPad Prism version 10 (GraphPad Software, San Diego, CA, USA).

## 3 Results

### 3.1 Study population and baseline characteristics

We identified 137 patients diagnosed with AL-CA or ATTR-CA, of whom 17 (12%) were excluded from the analysis due to limitations in image quality. One hundred twenty patients were included in the final analysis. TAPSE/PASP was available in all, whereas FAC/PASP and RVFWS/PASP could be assessed in 118 and 116 patients, respectively. The median age was 77 (IQR: 72–81) years, and 87% were male. A total of 103 patients (86%) were diagnosed with ATTR-CA, while the remaining 17 patients (14%) had AL-CA. The median values of TAPSE/PASP, FAC/PASP, and RVFWS/PASP were 0.41 mm/mmHg (IQR: 0.32–0.60), 0.88%/mmHg (IQR: 0.62–1.18), and 0.41%/mmHg (IQR: 0.29–0.63), respectively.

The study population was stratified based on the ROC-derived TAPSE/PASP cutoff of <0.30 mm/mmHg, demonstrating RV–PA uncoupling. While patients with RV–PA uncoupling tended to be older and had worse New York Heart Association functional class and a greater prevalence of atrial fibrillation and chronic kidney disease, these differences did not reach statistical significance. However, they showed a significantly higher use of loop diuretics (*p* = 0.017) and mineralocorticoid receptor antagonists (MRA) (*p* = 0.011), suggesting more advanced disease. In terms of biomarkers, the RV–PA uncoupling group had significantly elevated NT-proBNP (*p* < 0.001) and troponin T (*p* < 0.001), as well as higher bilirubin (*p* = 0.024), creatinine (*p* = 0.030), GGT (*p* = 0.037), and CRP/albumin ratio (*p* = 0.004). Echocardiographic assessment revealed that patients with RV–PA uncoupling had significantly worse LV systolic function, including lower LVEF and LVGLS. They also demonstrated worse RV function (lower TAPSE, FAC, RVFWS, and right ventricular global longitudinal strain (RVGLS); all *p* < 0.001), more pronounced PH (higher TRV and PASP; both *p* < 0.001), and greater left atrial enlargement (*p* = 0.010 for LAVI). Diastolic dysfunction was also more pronounced, with a significantly higher *E*/*A* ratio (*p* = 0.009), although other diastolic parameters showed no significant differences. Moderate tricuspid regurgitation (TR) was present in 34 patients (28%) and severe in 9 patients (8%) (Tables 1 and 2).

**Table 1:** Baseline characteristics of the study population stratified by RV–PA uncoupling (TAPSE/PASP <0.30 mm/mmHg).

Variable	All, *N* = 120	RV–PA uncoupling, *N* = 27	RV–PA coupling, *N* = 93	*p*-value
Demographic data				
Age, years	77 (72–81)	79 (76–81)	77 (73–80)	0.224
Male sex, *n* (%)	105 (87)	25 (93)	80 (82)	0.516
BMI, kg/m^2^	26.23 ± 0.63	26.59 ± 3.20	25.59 ± 3.53	0.195
BSA, m^2^	1.96 ± 0.16	1.99 ± 0.15	1.93 ± 0.16	0.079
SBP, mmHg	131.42 ± 23.34	128.46 ± 22.49	134.38 ± 24.07	0.283
DBP, mmHg	71.85 ± 11.34	71.96 ± 12.41	71.74 ± 10.22	0.930
Heart rate, bpm	76.08 ± 14.28	78.58 ± 14.28	73.58 ± 14.28	0.139
Amyloidosis type				
AL-CA, *n* (%)	17 (14)	10 (16)	7 (12)	0.645
Revised Mayo Staging for AL-CA	Stage I	1	0	0.073
	Stage II	6	0	
	Stage III	2	4	
	Stage IV	3	1	
ATTR-CA, *n* (%)	103 (86)	52 (84)	51 (88)	0.327
NAC staging for ATTR-CA	Stage I	4 (14)	38 (45)	0.026
	Stage II	12 (48)	31 (37)	
	Stage III	9 (36)	16 (19)	
NYHA functional class III or IV, *n* (%)	38 (32)	12 (44)	26 (28)	0.157
Comorbidities				
Hypertension, *n* (%)	78 (65)	16 (59)	62 (66)	0.499
Dyslipidaemia, *n* (%)	67 (56)	15 (56)	52 (56)	0.974
Diabetes, *n* (%)	28 (23)	7 (26)	21 (23)	0.797
CAD, *n* (%)	43 (36)	14 (52)	29 (31)	0.068
CKD, *n* (%)	76 (63)	20 (74)	56 (60)	0.257
Atrial fibrillation, *n* (%)	73 (61)	19 (70)	54 (58)	0.273
Pacemaker, *n* (%)	13 (11)	2 (7)	11 (12)	0.730
ICD, *n* (%)	5 (4)	1 (4)	4 (4)	0.890
CRT-D, *n* (%)	2 (2)	0 (0)	2 (2)	1.000
Medical therapy				
ACEi, *n* (%)	34 (28)	5 (19)	29 (31)	0.233
ARB, *n* (%)	35 (29)	9 (33)	26 (28)	0.634
ARNI, *n* (%)	10 (8)	3 (11)	7 (8)	0.692
Betablockers, *n* (%)	78 (65)	17 (63)	61 (66)	0.822
MRA, *n* (%)	41 (34)	15 (56)	26 (28)	0.011
SGLT2i, *n* (%)	39 (33)	12 (44)	27 (29)	0.163
Loop diuretics, *n* (%)	83 (69)	24 (89)	59 (63)	0.017

ACEi, angiotensin-converting enzyme inhibitors; AL-CA, light-chain cardiac amyloidosis; ARB, angiotensin receptor blockers; ARNI, angiotensin receptor neprilysin inhibitor; ATTR-CA, cardiac transthyretin amyloidosis; BMI, body mass index; bpm, beats per minute; BSA, body surface area; CAD, coronary artery disease; CKD, chronic kidney disease; CRT, cardiac resynchronization therapy; DBP, diastolic blood pressure; ICD, implantable cardioverter-defibrillator; MRA, mineralocorticoid receptor antagonists; NAC, National Amyloidosis Center; NYHA, New York Heart Association; SBP, systolic blood pressure; SGLT2i, sodium–glucose cotransporter-2 inhibitors.

**Table 2:** Laboratory and echocardiographic findings of the study population stratified by RV–PA uncoupling (TAPSE/PASP <0.30 mm/mmHg).

Variable	All, *N* = 120	RV–PA uncoupling, *N* = 27	RV–PA coupling, *N* = 93	*p-*value
Laboratory findings				
AST, U/L	31 (24–37)	29.5 (25–42)	31.5 (24–36)	0.575
ALT, U/L	25 (19–34)	23.5 (19–30)	26 (20–35)	0.291
Bilirubin, mg/dL	12 (9–18)	14.5 (11–23)	12 (9–16)	0.024
LDH, U/L	267.6 ± 90.6	291.75 ± 108.44	258.99 ± 82.57	0.129
AP, U/L	82 (69–110)	87.5 (71–121)	81.0 (68–108)	0.372
GGT, U/L	54.5 (32–112)	83.5 (41–198)	52 (30–83)	0.037
eGFR, mL/min	53.87 ± 21.02	47.44 ± 18.70	55.86 ± 21.39	0.069
Creatinine, μmol/L	106 (92–136)	118 (105–165)	103 (91–133)	0.030
NT-proBNP, ng/L	3,066 (1,236–5,610)	5,026 (3,163–11,331)	2,514 (964–4,249)	<0.001
Troponin T, ng/L	50 (31–76)	73 (56–113)	40 (29–66)	<0.001
Albumin, g/L	43.69 ± 0.68	40.74 ± 4.65	41.54 ± 6.08	0.567
CRP/albumin ratio	0.04 (0.02–0.1)	0.03 (0.02–0.06)	0.1 (0.04–0.14)	0.004
Echocardiography				
*E*/*A*	1.9 (0.99–2.72)	2.9 (2.68–3.6)	1.8 (0.98–2.6)	0.009
EDT, ms	209 (160–247)	202 (145–232)	210 (161–253)	0.451
*E*′ medial, cm/s	4.25 ± 0.25	3.97 ± 1.02	4.39 ± 1.29	0.167
*E*′ lateral, cm/s	5.37 ± 0.35	6.08 ± 2.96	5.66 ± 2.08	0.445
*E/E*′ medial	18.73 ± 1.09	23.82 ± 7.97	21.47 ± 7.84	0.247
*E/E*′ lateral	15.3 (11.5–21.8)	20 (12–22)	16 (12–21)	0.916
LVEF, %	48 ± 11	41 ± 13	50 ± 10	<0.001
LVGLS, %	−11.8 ± 4.2	−9.13 ± 3.36	−12.51 ± 4.12	<0.001
LVEDD, cm	4.74 ± 0.13	4.61 ± 0.59	4.60 ± 0.78	0.954
IVSD, cm	1.6 (1.4–1.9)	1.7 (1.5–2.0)	1.5 (1.4–1.9)	0.164
LVPW, cm	1.35 ± 0.07	1.54 ± 0.33	1.36 ± 0.34	0.014
RVWD, cm	0.97 ± 0.03	0.89 ± 0.21	0.93 ± 0.17	0.395
LVMI, g/m^2^	159.73 ± 43.31	181.44 ± 50.55	153.63 ± 39.25	0.004
RVEDD, cm	3.66 ± 0.10	3.89 ± 0.54	3.56 ± 0.59	0.009
TAPSE, mm	16 ± 5	10 ± 2	17 ± 4	<0.001
FAC, %	34 ± 10	24 ± 6	36 ± 9	<0.001
RVFWS, %	−16.0 ± 5.8	−11 ± 3.3	−17.7 ± 5.4	<0.001
RVGLS, %	−12.1 ± 4.7	−8.2 ± 2.5	−13.5 ± 4.5	<0.001
TRV, m/s	2.38 ± 0.11	2.87 ± 0.41	2.45 ± 0.45	<0.001
PASP, mmHg	33.85 ± 2.19	47.52 ± 7.94	34.42 ± 9.42	<0.001
LAVI, mL/m^2^	52.5 (45.1–65.1)	57 (49–73)	52 (44–63)	0.010
RA size, cm^2^	24.73 ± 1.21	26.98 ± 6.16	24.18 ± 7.07	0.066

AP, alkaline phosphatase; ALT, alanine aminotransferase; AST, aspartate aminotransferase; CRP, C-reactive protein; eGFR, estimated glomerular filtration rate; EDT, e-wave deceleration time; *E*/*A*, early diastolic velocity to late diastolic velocity ratio; *E/E*′ lateral, e-wave to early diastolic mitral annular velocity ratio (lateral); *E/E*′ medial, e-wave to early diastolic mitral annular velocity ratio (medial); *E*′ lateral, early diastolic mitral annular velocity (lateral); *E*′ medial, early diastolic mitral annular velocity (medial); FAC, %, fractional area change; GGT, gamma-glutamyl transferase; IVSD, interventricular septal thickness; LAVI, left atrial volume index; LDH, lactate dehydrogenase; LVEDD, left ventricular end-diastolic diameter; LVGLS, left ventricular global longitudinal strain; LVMI, left ventricular mass index; LVPW, left ventricular posterior wall thickness; LVEF, left ventricular ejection fraction; NT-proBNP, N-terminal pro B-type natriuretic peptide; PASP, pulmonary artery systolic pressure; RA, right atrial; RVEDD, right ventricular end-diastolic diameter; RVFWS, right ventricular free wall strain; RVGLS, right ventricular global longitudinal strain; RVWD, right ventricular wall thickness in diastole; TAPSE, tricuspid annular plane systolic excursion; TRV, tricuspid regurgitation velocity.

### 3.2 RV–PA uncoupling, all-cause mortality, and prognostic value of RV–PA coupling surrogates

Over a median follow-up period of 23 months (IQR: 15–34), the primary endpoint of all-cause mortality was observed in 25 patients, constituting 21% of the study population. When compared with long-term survivors, non-survivors had lower systolic blood pressure (SBP), higher levels of NT-proBNP and troponin T, worse renal and liver function, and lower serum albumin levels. In addition, non-survivors exhibited significantly worse systolic and diastolic LV function, as well as more impaired systolic RV function, including all analyzed RV and RV–PA parameters, although PA pressures did not differ (Supplementary Table S1).

**Figure 1:**
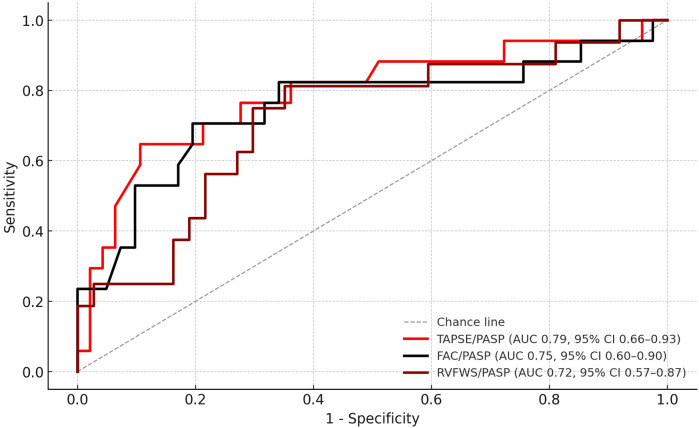
ROC curves for RV–PA coupling surrogates predicting 24-month all-cause mortality. ROC, receiver operating characteristic; RV–PA, right ventricular–pulmonary arterial; TAPSE, tricuspid annular plane systolic excursion; PASP, pulmonary artery systolic pressure; FAC, fractional area change; RVFWS, right ventricular free wall strain.

In continuous Cox analysis, lower values of all RV–PA coupling surrogates were independently associated with higher mortality (HR per +0.1 unit: TAPSE/PASP, 0.74, 95% CI: 0.59–0.93, *p* = 0.010; FAC/PASP, 0.87, 0.77–0.98, *p* = 0.018; RVFWS/PASP, 0.78, 0.63–0.97, *p* = 0.024). At the 24-month horizon, TAPSE/PASP demonstrated the strongest prognostic discrimination (AUC: 0.79, 95% CI: 0.66–0.91), compared with FAC/PASP (AUC: 0.75, 95% CI: 0.58–0.91) and RVFWS/PASP (AUC: 0.72, 95% CI: 0.52–0.87) (Figure 1). Pairwise AUC comparisons using DeLong's test indicated no statistically significant differences between surrogates (TAPSE/PASP vs. FAC/PASP, *p* = 0.42; TAPSE/PASP vs. RVFWS/PASP, *p* = 0.31; FAC/PASP vs. RVFWS/PASP, *p* = 0.58). At the same time horizon, uncorrected RV parameters showed numerically higher AUCs of 0.84 (TAPSE), 0.80 (FAC), and 0.74 (RVFWS) compared with afterload-adjusted equivalents. However, paired bootstrap ΔAUC: 95% CIs all overlapped zero (Supplementary Table S2). Optimal cutoffs by the Youden index were 0.30 for TAPSE/PASP (sensitivity 0.65, specificity 0.89), 0.705 for FAC/PASP (0.71, 0.80), and 0.391 for RVFWS/PASP (0.81, 0.65).

**Figure 2:**
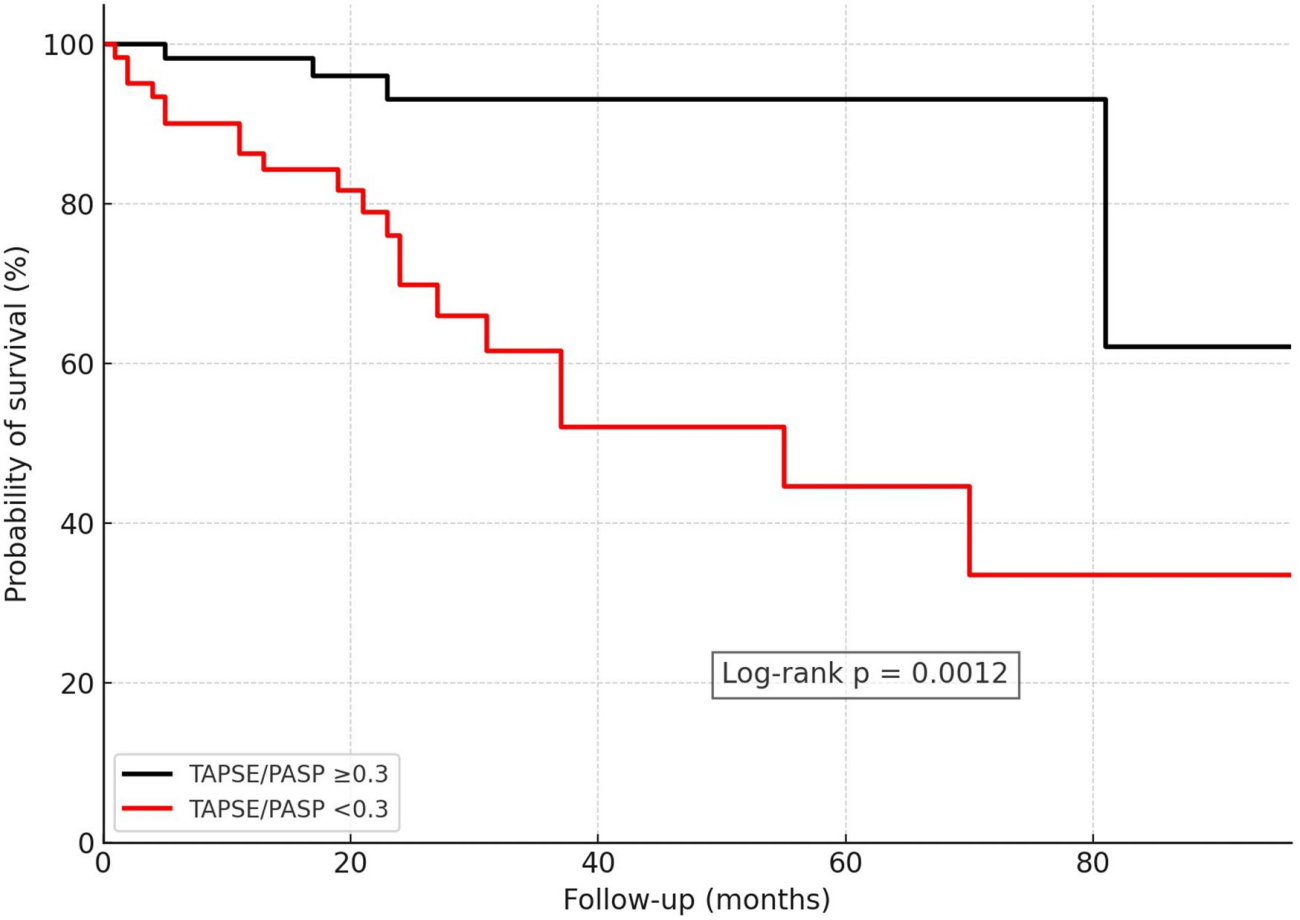
Kaplan–Meier survival analysis in CA patients stratified by RV–PA uncoupling using the ROC-derived optimal cutoff of TAPSE/PASP = 0.30 mm/mmHg. CA, cardiac amyloidosis; RV–PA, right ventricular–pulmonary arterial; TAPSE, tricuspid annular plane systolic excursion; PASP, pulmonary artery systolic pressure.

Kaplan–Meier analyses demonstrated clear separation of survival curves using these thresholds (log-rank *p* < 0.001 for TAPSE/PASP, *p* < 0.001 for FAC/PASP, *p* = 0.0048 for RVFWS/PASP; Figure 2). The number of patients at risk at 0, 12, and 24 months for all Kaplan–Meier analyses is reported in Supplementary Table S3.

Table 3 reports on the univariate Cox regression analysis of clinical, laboratory, and echocardiographic parameters associated with mortality. In univariable Cox regression, SBP, estimated glomerular filtration rate (eGFR), NT-proBNP, troponin T, albumin, LVEF, TAPSE, FAC, RVFWS, RVGLS, and all RV–PA coupling surrogates were significantly associated with mortality. Among the RV–PA coupling surrogates, all three (TAPSE/PASP, FAC/PASP, and RVFWS/PASP) were associated with mortality, but TAPSE/PASP provided the strongest and most robust prognostic discrimination across analytic approaches.

**Table 3:** Univariable Cox regression analysis for all-cause mortality.

Variable	HR (95% CI)	*p*-value
SBP, mmHg	0.965 (0.941–0.990)	0.006
eGFR, mL/min.	0.974 (0.956–0.992)	0.004
NT-proBNP per 100 ng/L	2.646 (1.761–3.978)	<0.001
cTroponinT, ng/L	1.017 (1.010–1.023)	<0.001
Albumin, g/L	0.879 (0.816–0.947)	<0.001
*E/E*′ lateral	1.041 (0.999–1.085)	0.056
LVEF (%)	0.970 (0.943–0.999)	0.041
TAPSE, mm	0.814 (0.736–0.901)	<0.001
FAC, %	0.910 (0.864–0.958)	<0.001
RVFWS, %	0.876 (0.801–0.957)	0.003
RVGLS, %	0.835 (0.742–0.939)	0.003
TAPSE/PASP, mm/mmHg (per +0.1)	0.742 (0.590–0.932)	0.010
FAC/PASP, %/mmHg (per +0.1)	0.865 (0.767–0.976)	0.018
RVFWS/PASP, %/mmHg (per +0.1)	0.780 (0.627–0.968)	0.024

*E/E*′, e-wave to early diastolic mitral annular velocity ratio; eGFR, estimated glomerular filtration rate; FAC, fractional area change; LVEF, left ventricular ejection fraction; PASP, pulmonary artery systolic pressure; RVFWS, right ventricular free wall strain; RVGLS, right ventricular global longitudinal strain; RV–PA, right ventricular to pulmonary artery coupling; SBP, systolic blood pressure; TAPSE, tricuspid annular plane systolic excursion.

## 4 Discussion

In this study, we analyzed the prevalence and prognostic significance of RV–PA uncoupling and compared the predictive value of different echocardiographic surrogates of RV–PA coupling in patients with CA. We demonstrated that RV–PA uncoupling, as evaluated by an ROC-derived TAPSE/PASP cutoff of 0.30 mm/mmHg, was strongly associated with increased all-cause mortality. TAPSE/PASP outperformed FAC/PASP and RVFWS/PASP, demonstrating the most robust surrogate of RV–PA uncoupling in CA. However, in direct AUC comparisons, the incremental prognostic value of coupling indices over conventional RV parameters was not statistically significant, as bootstrap ΔAUC 95% confidence intervals overlapped zero. This suggests that, within our dataset, coupling parameters did not consistently outperform their afterload-uncorrected RV parameters. Recently, there has been increasing evidence that the TAPSE/PASP ratio outperforms standard echocardiographic markers of RV function. While thresholds for the TAPSE/PASP ratio describing RV–PA uncoupling vary widely due to cohort stratification primarily using median values, there is clear evidence that TAPSE/PASP serves as a strong, independent predictor of mortality and adverse events across a broad spectrum of HF patients. RV–PA uncoupling, as assessed by the TAPSE/PASP ratio ranging from 0.39 to 0.55 mm/mmHg, emerged as an independent predictor of long-term mortality in patients with severe aortic stenosis undergoing transcatheter or surgical aortic valve repair (15–17). RV–PA uncoupling predicted adverse events in patients with HF independently across a wide range of LVEF. A TAPSE/PASP ratio <0.58 mm/mmHg in patients with HFrEF undergoing cardiac resynchronization therapy (CRT) was associated with higher mortality and a higher rate of HF hospitalization. In addition, it served as a predictor for CRT response (18). TAPSE/PASP ratio was prognostic in patients with acute decompensation and HFpEF (19). The analysis of RV–PA uncoupling in patients with CA has been limited. Tomasoni et al. (20) demonstrated that a TAPSE/PASP ratio <0.45 mm/mmHg was independently associated with a higher risk of all-cause mortality and HF hospitalization rates in patients with AL-CA and ATTR-CA during an 18-month median follow-up. Similarly, TAPSE/PASP identified RV–PA uncoupling with a ratio <0.45 mm/mmHg predicting short-term mortality at a median follow-up of 18 months in a cohort of patients with AL-CA and ATTR-CA (21). Our data corroborate the findings of the recent study by Palmiero et al. (22), which involved a similar patient cohort with comparable mortality rates. In this study, a TAPSE/PASP ratio of less than 0.30 mm/mmHg was associated with an increase in mortality, remaining an independent predictor of survival in multivariate analysis. Our study possesses the strength of a prolonged follow-up, with a median duration of 23 months, which expands upon previous findings and demonstrates that RV–PA uncoupling serves as a predictor of long-term outcomes.

While there is growing evidence supporting the use of TAPSE/PASP to address RV–PA coupling, there is a lack of data on alternative echocardiographic surrogates for RV–PA coupling. In a patient cohort with HFrEF and secondary PH, both TAPSE/PASP and FAC/PASP have exhibited similar correlations with Ees/Ea (23). The validation study by Tello et al. (14) on echocardiographic RV–PA surrogates revealed that among the surrogates, including TAPSE/PASP, FAC/mean PA pressure, RV area change/end-systolic area, and stroke volume/end-systolic area, only TAPSE/PASP showed a correlation with Ees/Ea. Considering that strain analysis provides a more precise assessment of the contractile state in comparison with traditional parameters like TAPSE, which focuses on a singular dimensional aspect of RV function, we hypothesized that RVFWS normalization with PASP would yield more robust prognostic data. In a substudy of the COAPT trial, RV–PA uncoupling was defined as RVFWS/PASP ≤0.5%/mmHg, which was associated with a twofold increased risk of death or hospitalization due to HF in patients with severe secondary mitral regurgitation (24). However, in our study, TAPSE/PASP outperformed numerically RVFWS/PASP in predicting long-term survival in the CA patient cohort, although RVFWS is a more sensitive parameter for RV function than conventional echocardiographic parameters.

It is still not well understood which RV amyloid burden leads to RV dysfunction and ultimately RV–PA uncoupling. Given the association between increasing RV amyloid, deterioration of RV structure, and impaired RV function, analysis of RV–PA coupling could potentially be utilized in therapy monitoring, particularly in the context of upcoming therapies aiming at amyloid depletion.

## 5 Limitations

This study was limited by its retrospective design. The analysis was feasible only with the available image data. The echocardiography images were not acquired in a dedicated core laboratory; however, the exams were performed in a standardized manner according to current guidelines for echocardiography by experienced examiners with high expertise. The non-invasive nature of the assessment of PASP provides only the estimated values, which can be considered a limitation, especially in cases lacking a satisfactory signal. In addition to TAPSE/PASP, validated as a reliable RV–PA coupling surrogate, we analyzed extended markers, including RVFWS/PASP and FAC/PASP, although these have not yet been sufficiently validated. We hypothesized that RVFWS/PASP would outperform TAPSE/PASP, as it is a more sensitive indicator for RV function than conventional echocardiographic parameters (25). Because the thresholds and performance metrics were derived and evaluated in the same dataset, they may be biased. We partially addressed this with bootstrap confidence intervals, but the lack of an external validation cohort limits their generalizability. Our ROC approach used a fixed 24-month horizon, which approximates time-dependent discrimination but does not implement full inverse probability of censoring weighted (IPCW)-based time-dependent ROC analysis, so future studies should confirm these cutoffs using IPCW or external validation cohorts. Significant tricuspid TR can influence the accuracy of TAPSE/PASP as a surrogate of RV–PA coupling. Although we reported the prevalence of moderate and severe TR and confirmed that exclusion of severe TR cases did not alter our findings, residual confounding cannot be fully excluded. Cardiovascular mortality as well as HF hospitalizations would serve as a more accurate endpoint; however, the retrospective study design made it challenging to verify these outcomes.

## 6 Conclusions

Impaired RV–PA coupling is strongly associated with increased mortality in patients with AL-CA and ATTR-CA. Among the evaluated surrogates, the TAPSE/PASP ratio provided the highest prognostic discrimination. TAPSE/PASP outperformed FAC/PASP and RVFWS/PASP numerically; however, it did not clearly outperform RV function parameters.

## Data Availability

The data that support the findings of this study are not publicly available due to ethical and legal restrictions. As this was a retrospective analysis, informed consent for data sharing was not obtained from the patients. Requests to access the datasets should be directed to berliner.dominik@mh-hannover.de.
