# Supplementary material for: Prognostic implications of right ventricular to pulmonary artery uncoupling in cardiac amyloidosis
Source: Front Cardiovasc Med. 2025 Sep 29;12:1653950. doi: 10.3389/fcvm.2025.1653950 (PMC12515962; doi:10.3389/fcvm.2025.1653950)
Supplement: Supplementary file 1 [file Table1.docx]

Supplementary Material

# Table S1. Baseline characteristics of the study population stratified by long-term survival

| **Variable** | **Non-survivors**  **N = 25** | **Survivors**  **N = 95** | **P value** |
| --- | --- | --- | --- |
| Age, years | 78 (72-81) | 77 (73-80) | 0.923 |
| Male sex (%) | 23 (92) | 82 (86) | 0.734 |
| SBP, mmHg | 117.55 ± 17.13 | 136.56 ± 23.71 | <0.001 |
| DBP, mmHg | 67.75 ± 10.65 | 72.70 ± 10.53 | 0.061 |
| AST, U/l | 29 (24-42) | 32 (25-37) | 0.710 |
| ALT, U/l | 21 (17-27) | 28 (20-35) | 0.018 |
| Bilirubin, mg/dl | 12 (8-20) | 13 (9-18) | 0.667 |
| LDH, U/l | 286.17 ± 105.01 | 261.35 ± 85.19 | 0.259 |
| AP, U/l | 91 (71-142) | 81 (67-107) | 0.116 |
| GGT, U/l | 79 (33-162) | 53 (31-90) | 0.186 |
| eGFR, ml/min. | 42.28 ± 22.64 | 57.12 ± 19.46 | 0.002 |
| NT-proBNP, ng/l | 8868 (3466-18531) | 2515 (970-4068) | <0.001 |
| Troponin T, ng/l | 79 (57-128) | 42 (30-65) | <0.001 |
| Albumin, g/l | 38.22 ± 6.63 | 42.38 ± 5.05 | 0.002 |
| CRP/Albumin ratio | 0.10 (0.03-0.46) | 0.04 (0.02-0.06) | 0.003 |
| E/A | 2.2 (0.93-2.85) | 1.9 (0.99-2.73) | 0.927 |
| E’ medial, ms | 3.61 ± 1.16 | 4.48 ± 1.22 | 0.004 |
| E’ lateral, ms | 4.29 ± 1.76 | 6.12 ± 2.25 | <0.001 |
| E/E’ medial | 25.00 ± 9.24 | 21.20 ± 7.73 | 0.059 |
| E/E’ lateral | 21.99 ± 8.33 | 16.84 ± 8.30 | 0.013 |
| LVEF, % | 43.50 ± 13.22 | 49.32 ± 10.18 | 0.019 |
| LVGLS, % | -10.34 ± 3.57 | -12.12 ± 4.28 | 0.064 |
| IVSd, cm | 1.5 (1.5-1.8) | 1.6 (1.4-1.9) | 0.785 |
| LVPW, cm | 1.41 ± 0.33 | 1.40 ± 0.35 | 0.968 |
| RVWD, cm | 0.94 ± 0.21 | 0.91 ± 0.17 | 0.583 |
| LVMI, g/m^2^ | 151.46 ± 31.87 | 161.93 ± 45.78 | 0.295 |
| TAPSE, mm | 12.65 ± 4.66 | 16.37 ± 4.40 | <0.001 |
| FAC, % | 28.04 ± 7.43 | 35.15 ± 9.96 | 0.001 |
| RVFWS, % | -12.99 ± 4.28 | -16.79 ± 5.88 | 0.005 |
| RVGLS, % | -9.60 ± 3.47 | -12.84 ± 4.73 | 0.003 |
| TRV, m/s | 2.60 ± 0.50 | 2.54 ± 0.47 | 0.579 |
| PASP, mmHg | 40.12 ± 10.97 | 36.64 ± 10.45 | 0.145 |
| TAPSE/PASP, mm/mm Hg | 0.26 (0.18-0.41) | 0.42 (0.33-0.6) | 0.001 |
| FAC/PASP, %/mm Hg | 0.65 (0.5-0.85) | 0.93 (0.68-1.23) | 0.007 |
| RVFWS/PASP, %/mm Hg | 0.33 (0.25-0.40) | 0.45 (0.31-0.64) | 0.018 |

AP = alkaline phosphatase; ALT = alanine aminotransferase; AST = aspartate aminotransferase; CRP = C-reactive protein; DBP = diastolic blood pressure; eGFR = estimated glomerular filtration rate; E/A = early diastolic velocity to late diastolic velocity ratio; E/E’ lateral = e-wave to early diastolic mitral annular velocity ratio (lateral); E/E’ medial = e-wave to early diastolic mitral annular velocity ratio (medial); E’ lateral = early diastolic mitral annular velocity (lateral); E’ medial = early diastolic mitral annular velocity (medial); FAC, % = fractional area change; GGT = gamma-glutamyl transferase; IVSD = interventricular septal thickness; LDH = lactate dehydrogenase; LVGLS = left ventricular global longitudinal strain; LVMI = left ventricular mass index; LVPW = left ventricular posterior wall thickness; LVEF = left ventricular ejection fraction; NT-proBNP = N-terminal pro B-type natriuretic peptide; PASP = pulmonary artery systolic pressure; RVFWS = right ventricular free wall strain; RVGLS = right ventricular global longitudinal strain; RVWD = right ventricular wall thickness in diastole; SBP = systolic blood pressure; TAPSE = tricuspid annular plane systolic excursion; TRV = tricuspid regurgitation velocity.
