# Supplementary material for: Prognostic implications of right ventricular to pulmonary artery uncoupling in cardiac amyloidosis
Source: Front Cardiovasc Med. 2025 Sep 29;12:1653950. doi: 10.3389/fcvm.2025.1653950 (PMC12515962; doi:10.3389/fcvm.2025.1653950)
Supplement: Supplementary file 2 [file Table2.docx]

Supplementary Material

**Table S2. Fixed-time ROC analyses at 24 months comparing uncorrected RV parameters with afterload-adjusted ratios (TAPSE/PASP, FAC/PASP and RVFWS/PASP)**

| **Parameter** | **AUC (95% CI)** | **Optimal cutoff** | **Sensitivity** | **Specificity** | **ΔAUC vs. afterload-adjusted (95% CI)** |
| --- | --- | --- | --- | --- | --- |
| TAPSE | 0.84 (0.72–0.94) | 17.5 mm | 0.78 | 0.82 | +0.053 (−0.004 to +0.128) |
| TAPSE/PASP | 0.79 (0.66–0.91) | 0.30 mm/mmHg | 0.65 | 0.89 |  |
| FAC | 0.80 (0.67–0.91) | 35 % | 0.75 | 0.77 | +0.048 (−0.006 to +0.119) |
| FAC/PASP | 0.75 (0.58–0.91) | 0.705 %/mmHg | 0.71 | 0.80 |  |
| RVFWS | 0.74 (0.60–0.88) | -18 % | 0.70 | 0.69 | +0.022 (−0.010 to +0.095) |
| RVFWS/PASP | 0.72 (0.52–0.87) | 0.391 %/mmHg | 0.81 | 0.65 |  |

FAC = fractional area change; PASP = pulmonary artery systolic pressure; RVFWS = right ventricular free wall strain; TAPSE = tricuspid annular plane systolic excursion.
