# Supplementary material for: Prognostic implications of right ventricular to pulmonary artery uncoupling in cardiac amyloidosis
Source: Front Cardiovasc Med. 2025 Sep 29;12:1653950. doi: 10.3389/fcvm.2025.1653950 (PMC12515962; doi:10.3389/fcvm.2025.1653950)
Supplement: Supplementary file 3 [file Table3.docx]

Supplementary Material

**Table S3. Numbers at risk for Kaplan–Meier analyses at 0, 12, and 24 months according to ROC-derived cutoffs of TAPSE/PASP, FAC/PASP and RVFWS/PASP**

| Parameter | Group | 0 months | 12 months | 24 months |
| --- | --- | --- | --- | --- |
| TAPSE/PASP | Coupled (TAPSE/PASP ≥0.300) | 93 | 78 | 47 |
| TAPSE/PASP | Uncoupled (TAPSE/PASP <0.300) | 27 | 20 | 10 |
| FAC/PASP | Coupled (FAC/PASP ≥0.705) | 73 | 63 | 38 |
| FAC/PASP | Uncoupled (FAC/PASP <0.705) | 39 | 27 | 13 |
| RVFWS/PASP | Coupled (RVFWS/PASP ≥0.391) | 53 | 46 | 27 |
| RVFWS/PASP | Uncoupled (RVFWS/PASP <0.391) | 53 | 38 | 19 |

FAC = fractional area change; PASP = pulmonary artery systolic pressure; RVFWS = right ventricular free wall strain; TAPSE = tricuspid annular plane systolic excursion.
